# On the Varieties of Alvine Discharges in Children

**Published:** 1852-07

**Authors:** 


*On the Varieties of Alvine Discharges in Children.* By Dr. Merei.

The intestinal discharges mentioned by the author are:

1. The *yellow* discharge. This is the regular kind of stool in infants. It is a mixture of intestinal secretions with bile. As children advance in age, and begin to take substantial food, the color of their regular discharge becomes more and more of a light brown color.

2. The *mucus* discharge. White mucus matter, more or less thick or liquid, and mixed with serum, sometimes with a proportion of bile. This discharge is preceded by but moderate pains, and frequently by no pains at all. It denotes a catarrhous, sub-inflammatory, or irritable state of the intestines, and is almost always of local, and not of sympathetic, origin; in general, it is not dangerous, and at its commencement is easily manageable by opiates, warm poultices, and convenient hygiene. If neglected, it becomes pertinacious and severe, and not seldom connected with swelling, softening, or granules of the mucous membrane, or ulceration of the follicles. If stripes of blood are mixed with the mucus, and pain be present, it denotes a higher degree of inflammation, in particular of the follicles. The highest development in this direction constitutes enteritis or colitis (dysentery.)

Sometimes we find among the mucus, consistent *plastic concretions* of a more or less tubular shape, similar to those of laryngeal croup, but larger in proportion to the volume of the intestines. This is the strongest degree of the catarrhous process, which I might term the *croup of the intestines.* Among the whole number of my little patients, which may be about 30,000, I met with this discharge perhaps only twenty or thirty times. The discharge is effected with very painful efforts at a stool.

3. The *serous.* In general, after more or less severe pains, the discharge takes place with a certain rigidity and noise, after which the pains lessen or subside. It consists of an abundant quantity of serous liquid, dirty whitish, yellowish, or greenish, as besides mucus, bile is the most common mixture with the serum. The serous diarrhœa is commonly the effect of rheumatism in the peritoneum, in the serous or fibrous membranes, or in the nerves of the intestines. I found in these cases the abdomen very hot. If a great deal of mucus and some blood are mixed with the serum, we may suspect parenchymatous enteritis; if the serous membrane alone enters into the state of acute inflammation, frequently transudation takes place on its free surface.

I have seen cases of profuse serous discharge, in a very short time, even in less than twenty-four hours, produce collapse and death, and in some of these instances necroscopy could not discover an adequate alteration either in the mucous or in the serous membrane.

The serous species of discharge is frequently merely a product of sympathetic secretion. I observed it sometimes connected with large transudations in the chest, and with chronic hydrocephalus.

Speaking in general, serous diarrhœa, if even arising from rheumatism, is more difficult to manage than the mucus. Very minute doses of calomel, with Dover's powder and mustard poultices, are frequently beneficial.

Pure serum, like rice-water, is a less favorable quality than the dirty white or yellowish. Dark brown serum frequently denotes a disorder in the portal system, present in some severe gastric or typhoid fevers, but I have seen a similar quality also in chronic affections of the brain, and very frequently in scrofulo-impetiginous children. This is worthy our attention, in particular if eczema or impetigo has disappeared from the head and face. This brown and fœtid discharge accompanies sometimes the commencement of chronic hydrocephalus. I treated it successfully, in this last case, with high but very diluted doses of iodide of potash.

4. *The green bilious discharge.* If pure bile, then the voided matter is, in general, not abundant. In young children, it is of a more yellowish than green color. The essential character of bile is, to be *of a greenish color* (in infants it is voided green) *at the very moment of its evacuation.* This kind of discharge is very frequently present in acute inflammatory and febrile affections; if dependent upon an affection of the brain, then we may find the color to be rather brown, and the abdomen retracted. If a similar source produces abundant serous-bilious discharges, then we find the abdomen much collapsed. But I must observe, acute affections of the brain are almost always connected with constipation; only in some cases of chronic hydrocephalus I met with the mentioned diarrhœa. Bilious discharge as arising from bilious fever, or from derangement of the liver, is rare in young children. In this case, the right hypochondrium will be more or less bloated up. We must be careful not to confound the green bilious discharge with the following.

5. The *discharge, like chopped eggs,* mixed with mucus, some clots of bile, and caseous coagula of indigested milk, or other kind of food, accompanied almost always by gripes and flatulence; its smell is disagreeably acid, and the whole matter, some minutes after being discharged and *exposed to the atmosphere, becomes green.* We know not exactly the chemical change which produces this coloration; it seems to be an oxydation of some of the elements. Then the essential character of this discharge is, that it is yellow at first, and becomes green by exposure to the atmosphere, while bile is green at the moment it comes out. I shall call this *the acid suburral discharge,* which is the most obvious before the sixth month of age, in particular if the sucking child takes, besides the milk, some farinaceous food. Practitioners commonly prescribe in this case rhubarb, with magnesia. For my part, I prefer, in tender infants, to rely more upon a convenient change in the diet, and as a remedy, aromatic frictions of the epigastrium, and internally bicarbonate of soda, dissolved in mint water.

6. The *bloody discharge.* Pure red blood is seldom discharged by children; in some rare cases I have seen half or one table-spoonfull come out, as the product of active congestion and hemorrhage. Very frequently, on the contrary, blood is combined with the mucus discharge, and in this case, if it is preceeded by pain, without tenderness, it denotes an inflammation in the upper parts of the intestinal tube, at least not near the rectum. Tenesmus signifies that the seat of the inflammation is in the lower parts of the colon, or in the rectum. This form is commonly called *dysentery,* not dangerous, if it is without bilious complication and fever, and if treated in its early stage with Dover's power, some doses of castor oil, and warm poultices; in a stronger degree, leeches at the anus; but if neglected in the commencement, it becomes dangerous to the life of the child. Professor Rokitansky, of Vienna, describes most exactly what he calls the "dysenteric process," in three gradual degrees of anatomical change. The highest degree, presenting a dirty red and gray marbled surface, with considerable thickening, granulation, and ulceration, I never saw in the tender age. Young children die before this stage is developed.

*Passive hemorrhage* of the intestines very seldom occurs in children. I have seen, however, some cases where, without adequate pain, a considerable quantity of dark thin blood was discharged. Lastly, we have seen in this town, with Mr. Wilson, a case in a child six years old, where, during the course of a gastro-typhoid fever, more than one pint of carbonized blood was discharged in two days. The case recovered. The boy is affected with an enlarged spleen.

Moderate quantities of red blood, discharged without pain, frequently occur, mixed with mucus, and are, without signification, sometimes even connected with the advance of recovery from gastric affections. This is the same case as with epistaxis.

Golding Bird and Simon state, as the result of chemical analysis, that some dark green stools of children owe this color to blood which has suffered a certain chemical change; but those chemical inquiries are not yet arrived at a satisfactory exactness; we do not even know exactly what kind of green discharges were the subject of these inquiries.

7. *Calomel stools.* Green, more or less thick, or mixed with serum, and in this case more abundant, produced by full doses of calomel. Calomel stools resemble bile, and contain much bile, but they contain also some particular chemical elements, which we do not exactly know. In many instances it happens that the calomel diarrhœa commences some days or weeks after the use of mercury, and we must be aware of this, and not confound it with the primary bilious discharge. In the former case, the region of the the liver is, in general, softer than in the latter. A clever practitioner will never try to stop directly, and with astringents, a green discharge, whatever be its origin and nature.

Calomel stools sometimes contain blood. After what I have seen in dissection, I incline to attribute this circumstance to a sub-inflammatory state, with superficial erosions of mucus membrane, which sometimes takes place in children after the continued use of calomel.

[The author states that he considers all these qualitative and physical distinctions of the discharges of children as very imperfect outlines of a sketch, which, by farther physical and chemical inquiry, can become corrected and perfected.]—*Ranking's Abstract.*

